# The Association of TLR2, TLR3, and TLR9 Gene Polymorphisms With Susceptibility to Talaromycosis Among Han Chinese AIDS Patients in Guangdong

**DOI:** 10.3389/fcimb.2021.625461

**Published:** 2021-03-11

**Authors:** Min Wang, Linghua Li, Saiyin Xiao, Wanshan Chen, Fengyu Hu, Feng Li, Pengle Guo, Xiejie Chen, Weiping Cai, Xiaoping Tang

**Affiliations:** ^1^ The First Affiliated Hospital, Jinan University, Guangzhou, China; ^2^ Guangzhou Eighth People’s Hospital, Guangzhou Medical University, Guangzhou, China; ^3^ Jiangmen Central Hospital, Affiliated Jiangmen Hospital of Sun Yat-sen University, Jiangmen, China

**Keywords:** Talaromycosis, *Talaromyces marneffei*, toll-like receptor, single nucleotide polymorphisms, susceptibility

## Abstract

**Background:**

Talaromycosis (TM) caused by *Talaromyces marneffei* (*T. marneffei*) is a growing public health concern. Although Toll-like receptor (TLR) genes play a critical role in the host defense against fungal infection, the influence of polymorphisms in these genes on the susceptibility of acquired immune deficiency syndrome (AIDS) patients to TM remains unknown. This study aims to uncover the associations of single nucleotide polymorphisms (SNPs) in TLR genes with TM susceptibility among patients with AIDS.

**Methods:**

Altogether 200 AIDS patients complicated with TM, 200 matched AIDS patients without TM, and 76 healthy controls (HCs) were enrolled in this case-control study. In total, 23 SNPs in the TLR2, TLR4, and TLR9 genes, which may influence the susceptibility of AIDS patients to TM, were checked by the time of flight mass spectrometry (TOF/MS) method among these Han Chinese subjects.

**Results:**

No significant differences in genotype or allele frequencies of selected SNPs were found among the TM group, Non-TM group, and HC group. Haplotype analysis also demonstrated no correlation of these SNPs with TM. However, subgroup analysis showed that the genotype TT and the T allele in TLR2 SNP rs1339 were more frequent in typical TM cases than controls (50.0 *vs.* 35.8%, 70.5 *vs.* 59.7%); the frequency of the GT genotype in TLR2 SNP rs7656411 was markedly higher in severe TM cases compared to controls (57.8 *vs.* 34.4%).

**Conclusion:**

Our results demonstrate a genetic connection of TLR2 SNPs rs1339 and rs7656411 with an increased susceptibility and severity of TM among Han Chinese populations.

## Introduction

Over the past 30 years, Talaromycosis (TM), a systemic mycosis caused by *Talaromyces marneffei* (*T. marneffei*), has become increasingly frequent among patients with acquired immune deficiency syndrome (AIDS), accounting for one of the most common invasive fungal diseases (IFDs) in Southeast Asia and southern China (He et al., 2020). Despite the significant progress attained in the management of this severe infection, its outcome remains unsatisfactory, with an approximate mortality of 10% annually, rendering it a leading infection-related cause of death among AIDS patients (Hanf et al., 2010). The remarkably high mortality rates owing to its challenges in prevention, diagnosis, and therapy, prompt physicians to focus on risk stratification and preemptive methods. This has inspired studying on novel individual prognostic signatures, especially gene signatures, to identify AIDS patients with an increased susceptibility to TM.

Toll-like receptors (TLRs) are the first identified and the best-studied family of pattern recognition receptors (PRRs), playing a crucial role in the first line of defense against invading pathogens and promoting adaptive immunity responses (Jungi and Werling, 2003; Albiger et al., 2007). Currently 11 members of the TLR superfamily (TLR1-11) have been identified in humans since the first discovery of TLR4 in late 1997 (Medzhitov et al., 1997; Zhang et al., 2004; Yarovinsky et al., 2005). These TLRs are expressed on the cell surface and/or in the endosome, of which four (TLR3, TLR7, TLR8, and TLR9) are expressed in the endosomal compartment (Kang and Lee, 2011). Endosomally localized TLRs share the responsibility of recognizing fungal, bacterial, and viral nucleic acids (Blasius and Beutler, 2010; Medvedev, 2013). On the other hand, the five TLRs (TLR1, TLR2, TLR4, TLR5, and TLR6) localized on the cell surface sense microbial surface associated pathogen associated molecular patterns (PAMPs) (Vijay, 2018). All TLRs except for TLR3 are able to signal through the adapter protein myeloid differentiation primary response 88 (MyD88) and then trigger nuclear factor kappa-B (NF-кB) activation and proinflammatory cytokine production, which have been revealed to play a pivotal role in protection against human infectious diseases (Kopp and Medzhitov, 2003; Yamamoto et al., 2003; Trejo-de la et al., 2014; Plato et al., 2015). Therefore, the role of single nucleotide polymorphisms (SNPs) within TLR genes, which may impair TLR functions, in host susceptibility to infectious diseases has received increasing attention. In particular, the TLR2 gene, TLR4 gene, and TLR9 gene have two exons, four exons, and two exons, which are located on chromosome 4q32, chromosome 9q33.1, and chromosome 3p21.2 in humans, respectively. Recent studies have demonstrated the importance of SNPs in the TLR2, TLR4, and TLR9 genes influencing the susceptibility and severity of several fungal diseases, but few studies have evaluated any associations with TM (Arbour et al., 2000; Balloy et al., 2005; Pamer, 2008; Ghosh and Stumhofer, 2013).

In the present study, a case-control model was conducted. The SNPs within the TLR2, TLR4, and TLR9 genes that may contribute to an increased susceptibility of AIDS patients to TM were checked by the time of flight mass spectrometry (TOF/MS) method. The relevance of TLR2 rs1339 and rs7656411 polymorphisms to the susceptibility of AIDS patients to TM provides a new understanding of the role of TLR in fungal infections and lays a theoretical basis for the risk prediction of TM.

## Method

### Research Subjects

In this study, a matched case-control design was carried out. A total of 476 Chinese Han adults who had resided in Guangdong Province for more than 1 year, were recruited from Guangzhou Eighth People’s Hospital from 2008 to 2015. Of these, 200 AIDS patients complicated with TM were enrolled as the case group (TM group), including 160 males and 40 females, with an age range of 19–69 years and a median age of 37.5 years. Another 200 AIDS patients with comparable gender and age, who did not develop TM, were enrolled in the control group (Non-TM group), including 163 males and 37 females, with an age range of 19–77 years and a median age of 39.5 years. In addition, 76 individuals who had a health examination in the same hospital during the same period and had no AIDS or other infectious diseases were enrolled as the healthy control group (HC group), including 53 males and 23 females, with an age range of 21–78 years and a median age of 38.6 years. The diagnosis of TM depends on the microscopic identification of the fungus with confirmation by culture as previously described (Ustianowski et al., 2008). All patients were confirmed to have AIDS and classified as C3, according to the AIDS diagnostic criteria of the US Centers for Disease Control and Prevention (CDC) 1993, enzyme linked immunosorbent assay (ELISA) and western blot confirmed HIV antibody positivity (Centers for Disease Control and Prevention, 1992).

A subgroup analysis was performed to account for differences in the severity of TM. Patients with TM were divided into two groups, severe cases and typical cases. Based on the severity of illness, the controls were chosen so that the severe or typical TM cases and their controls were matched by age and sex. The diagnostic criteria of severe TM referred to the Acute Physiology and Chronic Health Evaluation II and sequential organ failure assessment scoring systems (Hosseini and Ramazani, 2016), should be in line with at least one of the following characteristics: platelet count below 30 × 10^9^/L, mental disorders, septic shock, respiratory failure, and multiple organ damage.

This project was approved by the ethics committee of the Guangzhou Eighth People’s Hospital (GZ8H20171390), and informed consent was obtained from every subject recruited in the study.

### Sample Preparation and DNA Extraction

Peripheral venous blood (10 ml) was collected from each subject with an EDTA-K2 anticoagulation vacuum blood collection tube. Genome DNA of peripheral venous blood from all subjects was isolated by using the QIAamp DNA Mini Kit according to the manufacturer’s protocol. The purity and concentration of DNA were detected by an ultraviolet spectrophotometer and then frozen at −20°C until testing.

### Polymorphism Genotyping

The candidate polymorphisms that have previously been reported to be associated with infectious diseases in the literature, or identified minimal haplotype-tagging SNPs from the NCBI GenBank, dbSNP, and HapMap databases were genotyped after comprehensive evaluation. A total of 23 SNPs in TLR2, TLR4, and TLR9 were identified with the use of a TOF/MS method, with appropriate PCR primers as shown in **Supplementary Table 1**. The PCR system was a volume of 5 µl solution, which included substrate template DNA (0.01 µg), 0.5 µl forward primer (0.5 µmol/L), 0.5 µl reverse primer (0.5 µmol/L), 2.5 µl Master Mix, and the corresponding buffer solution. The PCR amplification conditions were as follows: an initial denaturation at 94°C for 15 min, followed by 45 cycles of 94°C for 20 s, 56°C for 30 s, and 72°C for 1 min and a final extension at 72°C for 3 min.

### Statistical Analysis

Pearson’s-chi-square and Fisher’s exact tests were applied to calculate and compare categorical data of clinical variables, and genotype and allele frequencies. All polymorphisms were tested with the Hardy-Weinberg equilibrium (HWE). Plink software was used to analyze linkage disequilibrium (LD) and haplotype analysis. Statistical analyses were conducted using the SPSS 13.0 software package. A P value less than 0.05 was regarded as statistically significant.

## Results

### Baseline Characteristics of the Subjects

A total of 200 TM patients, 200 non-TM patients, and 76 healthy controls were included in the study. The clinical characteristics of the patients, including age, sex, CD4+ T cell count, and prevalence of hepatitis or other opportunistic infections, were collected and compared (**Table 1**). Age and sex were not significantly different among the three groups. Similarly, there was no difference in the CD4+ T cell count or prevalence of actual hepatitis or other opportunistic infections (OIs) between patients with TM and non-TM patients. Furthermore, 200 AIDS patients with TM were divided into 134 typical cases and 66 severe cases for further analysis. The typical cases or severe cases and their controls were frequency matched by sex and age, as well as CD4+ T cell count and prevalence of other opportunistic infections, as shown in **Table 2**.

**Table 1:** Characteristics of the subjects selected for genotyping.

Characteristics	TM group (n = 200)	Non-TM group (n = 200)	HC group (n = 76)	χ^2^ value	*P*-value
Gender				4.48	0.11
Male	160 (80.0%)	162 (81.0%)	53 (69.7%)		
Female	40 (20.0%)	38 (19.0%)	23 (30.3%)		
Age (years)				2.59	0.63
18–45	157 (78.5%)	148 (74.0%)	54 (71.1%)		
46–65	41 (20.5%)	49 (24.5%)	20 (26.3%)		
≥66	2 (1.0%)	3 (1.5%)	2 (2.6%)		
CD4^+^ T cell count (cells/ul)				0.05	0.83
0–50	189 (94.5%)	188 (94.0%)	NA		
51–100	11 (5.5%)	12 (6.0%)	NA		
Acute hepatitis co-infection				2.62	0.11
Yes	34 (17.0%)	47 (28.5%)	NA		
No	166 (83.0%)	153 (71.5%)	NA		
Other OIs co-infection				NA	NA
Yes	200 (100.0%)	200 (100.0%)	NA		
No	0 (0.0%)	0 (0.0%)	NA		

TM group, AIDS patients with talaromycosis; Non-TM group, AIDS patients without talaromycosis; HC group, healthy controls group; OIs, opportunistic infections; NA, not applicable.

**Table 2:** Characteristics of the typical and severe TM patients.

Characteristics	Typical cases (n = 134)	Matched controls (n = 134)	χ2 value	P-value	Severe cases (n = 66)	Matched controls (n = 66)	χ^2^ value	*P*-value
Gender			0.24	0.62			0	1
Male	110 (82.1%)	113 (84.3%)			50 (75.8%)	50 (75.8%)		
Female	24 (17.9%)	21 (15.7%)			16 (24.2%)	16 (24.2%)		
Age (years)			0.22	0.90			3.42	0.07
18–45	102 (76.1%)	102 (76.1%)			55 (83.3%)	46 (69.7%)		
46–65	30 (22.4%)	29 (21.6%)			11 (16.7%)	20 (30.3%)		
≥66	2 (1.5%)	3 (2.3%)			0 (0.0%)	0 (0.0%)		
CD4^+^ T cell count (cells/ul)			0.05	0.82			0.34	0.56
0–50	124 (92.5%)	123 (91.8%)			65 (98.5%)	64 (97.0%)		
51–100	10 (7.5%)	11 (8.2%)			1 (1.5%)	2 (3.0%)		
Acute hepatitis co-infection			0.98	0.32			2.88	0.09
Yes	30 (22.4%)	37 (27.6%)			4 (6.1%)	10 (15.2%)		
No	104 (77.6%)	97 (72.4%)			62 (93.9%)	56 (84.8%)		
Other OIs co-infection			NA	NA			NA	NA
Yes	134 (100.0%)	134 (100.0%)			66 (100.0%)	66 (100.0%)		
No	0 (0.0%)	0 (0.0%)			0 (0.0%)	0 (0.0%)		

Typical cases, AIDS patients with typical talaromycosis; Severe cases, AIDS patients with severe talaromycosis; OIs, opportunistic infections.

### Association Between TLR2, TLR4, and TLR9 Polymorphisms and TM Susceptibility

In total, 23 candidate polymorphisms, all present in the coding region of the TLRs, were selected for analysis: 9 SNPs in the TLR2 gene (rs11935252, rs11938228, rs121917864, rs1339, rs3804099, rs5743708, rs76112010, rs7656411, and rs7682814), 12 SNPs in the TLR4 gene (rs10116253, rs11536878, rs11536889, rs11536891, rs1554973, rs1927911, rs2737191, rs4986790, rs4986791, rs5030728, rs7037117, and rs7856729), and 2 SNPs in the TLR9 gene (rs187084 and rs352140). In detail, 3 of the 9 genotyped TLR2 SNPs (rs121917864, rs5743708, and rs7682814) and 4 of the 12 genotyped TLR4 SNPs (rs2737191, rs4986790, rs4986791, and rs5030728) showed no polymorphisms in all the subjects, so only 16 SNPs were eligible for the subsequent analysis. All these SNPs were in Hardy-Weinberg equilibrium in controls except for the two TLR4 SNPs rs10116253 and rs7037117.

To study associations of TLR2, TLR4, and TLR9 polymorphisms with the risk of TM, the frequency distributions of genotypes and alleles were assessed. Overall, 13 of the 14 SNPs showed no significant differences in genotype or allele frequencies among the TM group, non-TM group, and HC group (**Table 3**). In particular, analysis of the TLR4 SNP rs11536889 demonstrated a statistically significant difference in the genotype distribution among the three groups, but not in the allele frequencies. When patients with TM were compared to control patients without TM, no significant difference in genotype or allele frequencies of the TLR4 SNP rs11536889 was observed (data not shown). Among the study subjects, the susceptibility of AIDS patients to TM seems to be independent of the selected TLR2, TLR4, and TLR9 polymorphisms.

**Table 3:** Allele and genotype frequencies of TLR2, TLR4, and TLR9 SNPs in the subjects.

Gene	SNP ID	Genotype or allele	TM group (n = 200)	Non-TM group (n = 200)	HC group (n = 76)	Missing	χ^2^ value	*P*-value
TLR2	rs11935252	Genotype				0	0.54	0.97
		A/A	122 (61.0%)	124 (62.0%)	44 (57.9%)			
		G/A	69 (34.5%)	67 (33.5%)	29 (38.2%)			
		G/G	9 (4.5%)	9 (4.5%)	3 (3.9%)			
		Allele					0.204	0.90
		A	313 (78.2%)	315 (78.7%)	117 (77.0%)			
		G	87 (21.8%)	85 (21.3%)	35 (23.0%)			
	rs11938228	Genotype				10	1.42	0.84
		C/C	68 (34.8%)	72 (36.9%)	24 (31.6%)			
		C/A	98 (50.3%)	96 (49.2%)	43 (56.6%)			
		A/A	29 (14.9%)	27 (13.9%)	9 (11.8%)			
		Allele					0.24	0.89
		C	234 (60.0%)	240 (61.5%)	91 (59.9%)			
		A	156 (40.0%)	150 (38.5%)	61 (40.1%)			
	rs1339	Genotype				0	4.30	0.37
		T/T	93 (46.5%)	80 (40.0%)	34 (44.7%)			
		T/C	88 (44.0%)	91 (45.5%)	36 (47.4%)			
		C/C	19 (9.5%)	29 (14.5%)	6 (7.9%)			
		Allele					3.40	0.18
		T	274 (68.5%)	251 (62.8%)	104 (68.4%)			
		C	126 (31.5%)	149 (37.2%)	48 (31.6%)			
	rs3804099	Genotype				4	3.38	0.50
		T/T	106 (53.5%)	109 (55.0%)	40 (52.6%)			
		T/C	73 (36.9%)	76 (38.4%)	33 (43.4%)			
		C/C	19 (9.6%)	13 (6.6%)	3 (4.0%)			
		Allele					0.48	0.79
		T	289 (72.3%)	294 (74.2%)	113 (74.3%)			
		C	111 (27.7%)	102 (25.8%)	39 (25.7%)			
	rs76112010	Genotype				0	3.51	0.48
		G/G	105 (52.5%)	100 (50.0%)	37 (48.7%)			
		G/A	80 (40.0%)	78 (39.0%)	35 (46.0%)			
		A/A	15 (7.5%)	22 (11.0%)	4 (5.3%)			
		Allele					0.91	0.64
		G	290 (72.5%)	278 (69.5%)	109 (71.7%)			
		A	110 (27.5%)	122 (30.5%)	43 (28.3%)			
	rs7656411	Genotype				12	1.98	0.74
		G/G	41 (21.1%)	40 (20.6%)	15 (20.0%)			
		G/T	99 (51.1%)	91 (46.9%)	41 (54.7%)			
		T/T	54 (27.8%)	63 (32.5%)	19 (25.3%)			
		Allele						
		G	181 (46.6%)	171 (44.1%)	71 (47.3%)			
		T	207 (53.4%)	217 (55.9%)	79 (52.7%)			
TLR4	rs11536878	Genotype				0	0.07	0.97
		C/C	157 (78.5%)	159 (79.5%)	61 (80.3%)			
		C/A	41 (20.5%)	39 (19.5%)	15 (19.7%)			
		A/A	2 (1.0%)	2 (1.0%)	0 (0.0%)			
		Allele					0.22	0.90
		C	355 (88.7%)	357 (89.2%)	137 (90.1%)			
		A	45 (11.3%)	43 (10.8%)	15 (9.9%)			
	rs11536889	Genotype				0	11.2	**0.02**
		C/C	11 (5.5%)	18 (9.0%)	14 (18.4%)			
		C/G	87 (43.5%)	84 (42.0%)	29 (38.2%)			
		G/G	102 (51.0%)	98 (49.0%)	33 (43.4%)			
		Allele					5.51	0.06
		C	109 (27.2%)	120 (30.0%)	57 (37.5%)			
		G	291 (72.8%)	280 (70.0%)	95 (62.5%)			
	rs11536891	Genotype				0	0.81	0.67
		C/C	3 (1.5%)	2 (1.0%)	0 (0.0%)				
		C/T	34 (17.0%)	28 (14.0%)	11 (14.5%)			
		T/T	163 (81.5%)	170 (85.0%)	65 (85.5%)			
		Allele					1.50	0.47
		C	40 (10.0%)	32 (8.0%)	11 (7.2%)			
		T	360 (90.0%)	368 (92.0%)	141 (92.8%)			
	rs1554973	Genotype				0	3.50	0.17
		C/C	4 (2.0%)	4 (2.0%)	1 (1.3%)			
		C/T	51 (25.5%)	41 (20.5%)	12 (15.8%)			
		T/T	145 (72.5%)	155 (77.5%)	63 (82.9%)			
		Allele					3.22	0.20
		C	59 (14.7%)	49 (12.2%)	14 (9.2%)			
		T	341 (85.3%)	351 (87.8%)	138 (90.8%)			
	rs1927911	Genotype				4	2.06	0.73
		A/A	16 (8.1%)	18 (9.1%)	5 (6.6%)			
		A/G	89 (44.9%)	96 (48.5%)	32 (42.1%)			
		G/G	93 (47%)	84 (42.4%)	39 (51.3%)			
		Allele					1.82	0.40
		A	121 (30.6%)	132 (33.3%)	42 (27.6%)			
		G	275 (69.4%)	264 (66.7%)	110 (72.4%)			
	rs7856729	Genotype				0	1.09	0.58
		G/G	162 (81.0%)	170 (85.0%)	65 (85.5%)			
		G/T	35 (17.5%)	28 (14.0%)	11 (14.5%)			
		T/T	3 (1.5%)	2 (1.0%)	0 (0.0%)			
		Allele					1.83	0.40
		G	359 (89.7%)	368 (92.0%)	141 (92.8%)			
		T	41 (10.3%)	32 (8.0%)	11 (7.2%)			
TLR9	rs187084	Genotype				2	3.62	0.46
		AA	74 (37.2%)	89 (44.7%)	36 (47.4%)			
		AG	99 (49.7%)	87 (43.7%)	33 (43.4%)			
		GG	26 (13.1%)	23 (11.6%)	7 (9.2%)			
		Allele					3.06	0.22
		A	247 (62.1%)	265 (66.6%)	105 (69.1%)			
		G	151 (37.9%)	133 (33.4%)	47 (30.9%)			
	rs352140	Genotype				2	2.76	0.60
		CC	77 (38.7%)	92 (46.2%)	35 (46.1%)			
		CT	97 (48.7%)	87 (43.7%)	33 (43.4%)			
		TT	25 (12.6%)	20 (10.1%)	8 (10.5%)			
		Allele					2.33	0.31
		C	251 (63.2%)	271 (68.1%)	103 (67.8%)			
		T	147 (36.8%)	127 (31.9%)	49 (32.2%)			

TM group, AIDS patients withTalaromycosis; Non-TM group, AIDS patients without talaromycosis; HC group, healthy controls group; Missing, genotyping failure; TLR, Toll-like receptor; SNP, single nucleotide polymorphism. In bold: P value less than 0.05 and is regarded as statistically significant.

On the other hand, when patients with TM were divided into typical cases and severe cases and compared to matched control patients, significant differences in genotype and allele frequencies of the TLR2 SNPs were observed (**Table 4**). The subgroup analysis demonstrated that the genotype TT and allele T in TLR2 SNP rs1339 were more frequent in typical cases than in controls (50.0 *vs.* 35.8%, 70.5 *vs.* 59.7%). In addition, the frequency of the GT genotype in the TLR2 SNP rs7656411 was found to be markedly higher in severe cases than in controls (57.8 *vs.* 34.4%). Moreover, the genotype and allele frequencies of selected SNPs were similar between patients with typical and severe TM (data not shown). The above results revealed that TLR2 SNPs rs1339 and rs7656411 may contribute to an increased susceptibility of AIDS patients to TM, and the GT genotype of TLR2 SNP rs7656411 may be implicated in the risk of severe TM among AIDS patients.

**Table 4:** Correlation between TLR2, TLR4, and TLR9 SNPs and risk of typical or severe TM.

Gene	SNP ID	Typical cases *vs* matched controls				Severe cases *vs* matched controls				Typical cases *vs* severe cases			
		Genotype		Allele		Genotype		Allele		Genotype		Allele	
		*χ²* value	*P* value	*χ²* value	*P* value	*χ²* value	*P* value	*χ²* value	*P* value	*χ²* value	P value	χ² value	P value
TLR2	rs11935252	1.29	0.52	0.91	0.34	1.20	0.57	1.07	0.30	0.78	0.68	0.49	0.49
	rs11938228	2.05	0.36	2.04	0.15	1.92	0.38	1.66	0.20	2.15	0.34	1.43	0.23
	rs1339	6.76	**0.03**	6.91	**0.01**	1.22	0.54	0.61	0.43	2.00	0.37	1.54	0.22
	rs3804099	0.97	0.62	0.24	0.62	0.34	0.84	0.31	0.58	0.99	0.61	0.06	0.81
	rs76112010	4.66	0.10	4.66	0.10	5.46	0.07	5.46	0.07	3.15	0.21	1.84	0.18
	rs7656411	3.47	0.18	2.24	0.13	8.39	**0.02**	0.77	0.38	3.12	0.21	1.04	0.31
TLR4	rs11536878	0.34	0.84	0.08	0.78	1.16	0.56	0.00	1.00	1.01	0.60	0.08	0.78
	rs11536889	2.51	0.29	1.81	0.18	0.78	0.68	0.25	0.62	1.64	0.44	1.44	0.23
	rs11536891	0.64	0.73	0.61	0.45	0.48	0.82	0.37	0.54	0.51	0.78	0.41	0.52
	rs1554973	0.38	0.86	0.26	0.61	3.01	0.24	1.16	0.28	0.64	0.73	0.21	0.65
	rs1927911	0.59	0.74	0.31	0.58	1.13	0.57	0.46	0.50	1.13	0.74	0.75	0.39
	rs7856729	0.64	0.73	0.61	0.44	0.82	0.67	0.64	0.42	0.95	0.62	0.75	0.39
TLR9	rs187084	3.20	0.20	2.63	0.11	0.15	0.93	0.00	1.00	3.39	0.18	1.24	0.27
	rs352140	2.42	0.30	2.11	0.30	0.28	0.87	0.28	0.60	2.50	0.29	0.69	0.41

Typical cases, AIDS patients with typical talaromycosis; Severe cases, AIDS patients with severe talaromycosis; TLR, Toll-like receptor; SNP, single nucleotide polymorphism. In bold: P value less than 0.05 and is regarded as statistically significant.

**Figure 1:**
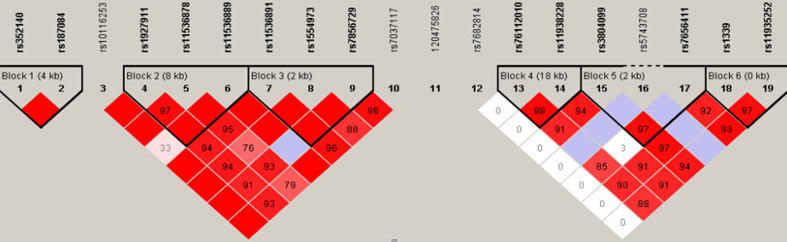
Haplotype analyses among TLR2, TLR4, and TLR9 SNPs. The TLR9 (chromosome 3), TLR4 (chromosome 9), and TLR2 (chromosome 4) genes cover 4, 10, and 20 kb, respectively. The location of the genotyped SNPs is indicated with vertical lines. Pairwise linkage disequilibrium (LD) (D’) that was observed in the research population (n = 476) is also depicted. The figure shows R^2^ values for pairwise comparison of the different polymorphisms. R^2^ = 0 represents no LD correlation; R^2^ = 1 represents high (maximum) LD correlation. TLR, Toll-like receptor; SNP, single nucleotide polymorphism.

Moreover, TLR haplotypes were constructed and analyzed using the Haploview program. Fourteen of the 19 SNPs were defined into six haplotype blocks (**Figure 1**). Haplotypes with frequencies greater than 0.01 were considered eligible for analysis; but, none of them were closely associated with TM.

## Discussion

This study is the first to identify a connection of TM susceptibility with the specific polymorphism (rs1339 and rs7656411) rather than all TLR2 SNPs and the first to reveal a noticeable association between the GT genotype of TLR2 SNP rs7656411 and the severity of TM. Our findings suggest that genetic polymorphisms in TLR2 may play a critical role in the occurrence and pathogenesis of TM, and could also serve as a feature underlying the susceptibility and severity of TM among Han Chinese populations.

Associations with the TLR2 gene have been established for several fungal infections (Villamón et al., 2004; Balloy et al., 2005; Woehrle et al., 2008; Kastelijn et al., 2010; Wong et al., 2010). For example, TLR2 is considered one of the major receptors for the opportunistic fungus *Aspergillus fumigatus*, because of the survival difference between TLR2 knockout mice and wild-type mice upon induction of invasive pulmonary aspergillosis (Balloy et al., 2005). Additionally, Villamón and coworkers showed that TLR2-deficient mice are more susceptible to systemic challenge with *Candida albicans* (Villamón et al., 2004). However, the influence of TLR SNPs on human diseases, especially fungal diseases, has only recently been investigated. To our knowledge, this is the first study to confirm a genetic association between TLR2 SNPs such as rs1339 and rs7656411 and the susceptibility and severity of TM in humans. The TLR2 SNPs rs1339 and rs7656411 have been previously reported to be associated with susceptibility to leprosy and bronchiolitis obliterans, respectively (Kastelijn et al., 2010; Wong et al., 2010). Moreover, a similar association between TLR2 polymorphism and Candida sepsis has been observed in humans (Woehrle et al., 2008). One of the possible explanations is the attenuated responses of TLR SNPs to TLR ligands, although the exact molecular mechanisms through which TLR polymorphisms contribute to TM pathogenesis remain unknown (Schröder and Schumann, 2005). It is presumed that the polymorphism changes the electrostatic potential of the Toll/interleukin-1 receptor (TIR) domain of TLR2, resulting in impaired agonist-inducible tyrosine phosphorylation of TLR2, TLR2-TLR6 dimerization, recruitment of MyD88, and suppression of transcription factors such as NF-κB activation and secretion of important inflammatory mediators (Xiong et al., 2012). These changes have been reported to exert a significant effect on the clearance of infections, and often explain the different outcomes of the host to a certain infectious disease (Bellocchio et al., 2004). Moreover, Jiang et al. exhibited a significant relationship between TLR2 SNPs and susceptibility to non-HIV cryptococcal meningitis (CM) and found that non-HIV CM patients carrying the TLR2 rs3804099 C/T genotype had significantly lower levels of 12 cerebrospinal fluid (CSF) cytokines, predominantly proinflammatory cytokines and chemokines, suggesting that the rs3804099 C/T genotype may downregulate immune responses (Jiang et al., 2018). Thus, certain SNPs in TLR2 may increase the susceptibility of AIDS patients not only to TM, but also to acute hepatitis and other opportunistic coinfections. Interestingly, Carvalho et al. showed a potentially protective role of TLR2 SNPs in allergic bronchopulmonary aspergillosis (ABPA) (Carvalho et al., 2008). Consequently, the functional relevance of TLR2 SNPs should be investigated in more detail.

Recent studies on the role of TLR4 SNPs in fungal diseases have yielded conflicting results. For instance, Carvalho et al. suggested a positive association between Asp299Gly (rs4986790) TLR4 and chronic cavitary pulmonary aspergillosis (CCPA) (Carvalho et al., 2008). Bochud et al. showed that the common cosegregating TLR4 non-synonymous SNPs, Asp299Gly (rs4986790) and Thr399Ile (rs4986791) in unrelated donors were associated with an increased risk of invasive aspergillosis (IA) among recipients of hematopoietic cell transplants (Bochud et al., 2008). Koldehoff et al. found Asp299Gly and Thr399Ile to be highly associated with an increased susceptibility of transplant recipients to IA whenever present in donors or recipients of allogeneic stem cell transplantation (ASCT) (Koldehoff et al., 2013). In contrast, the significant association between IA and Asp299Gly and Thr399Ile was unable to be observed in a smaller study by Kesh and coworkers (Kesh et al., 2005). Interestingly, our results demonstrated no TLR4 Asp299Gly and Thr399Ile polymorphisms in all Han Chinese subjects from Guangdong, indicating that the two SNPs may play a minor role in increasing the susceptibility of ethnic Chinese populations to TM. Similar findings were also described for other Asian ethnic populations by several previous studies (Hang et al., 2004; Guo et al., 2005; Nakada et al., 2005; Kim et al., 2012). It is likely that the distribution of TLR4 gene polymorphisms has ethnic differences due to local evolutionary pressures by infection (Ferwerda et al., 2007). Therefore, although the association of TLR4 SNPs with TM susceptibility cannot be observed in our study on Han Chinese populations, comprehensive studies of a larger number of patients with different ethnic backgrounds are required to draw definitive conclusions.

TLR9 is the only TLR in humans that senses unmethylated cytosine-phosphate-guanine (CpG) motifs prevalent in bacterial or viral single stranded DNA (Hemmi et al., 2000). A number of SNPs for TLR9 have been identified, and two of these SNPs, rs187084 (A1486G) and rs352140 (C2848T), are often reported to influence susceptibility or resistance to infections (Hemmi et al., 2000; Ferwerda et al., 2007). TLR9 A1486G carriers have been found to be associated with a decreased risk of *Acinetobacter baumannii* in a Chinese population (He et al., 2016), and the C2848T polymorphism is significantly positively related to neonatal severe hepatitis among Chinese newborns (Qiu et al., 2018). Moreover, allele C on T-1237C (TLR9) was associated with susceptibility to allergic bronchopulmonary aspergillosis (Carvalho et al., 2008). A Japanese study on systemic lupus erythematosus (SLE) showed that a C allele at position −1486 has the ability to downregulate TLR9 expression (Tao et al., 2007), indicating that C allele carriers may be susceptible to diseases associated with the TLR9 gene. In contrast, a study reported by Plantinga et al. showed no associations of SNPs in TLR2, TLR4, or TLR9 with susceptibility to candidemia (Plantinga et al., 2012). Our study also demonstrated no obvious associations of the two TLR9 SNPs with TM susceptibility among Han Chinese populations. Although the cause of this difference is unclear, the role of TLR9 polymorphisms in TM susceptibility cannot be completely excluded.

Overall, this study represented the first analysis evaluating whether TLR2, TLR4, and TLR9 could contribute to regulating the susceptibility or severity of TM among the Chinese Han population. Several limitations could not be ignored. First, the sample size was relatively limited in our cohort. Some genotypes of SNPs have low frequencies, which may restrict statistical power. Second, the studied subjects were all of Chinese Han ethnicities, so the results might not be well applicable to other groups due to ethnic differences. Third, all of the detected TLR SNPs are located in the coding region. Other SNPs present in the non-coding region of these TLRs may also be potential targets. Finally, only the superficial correlation between TLR SNPs and susceptibility to TM was investigated, and the potential mechanisms need further study.

In conclusion, the results of this study indicated that TLR2 SNPs such as rs1339 and rs7656411 were associated with an increased susceptibility and severity of TM among Han Chinese populations. Our findings not only imply the importance of TLR2 in natural immunity against fungi but also provide a theoretical basis for the risk and severity prediction of TM.

## Data Availability Statement

The original contributions presented in the study are included in the article/**Supplementary Material**. Further inquiries can be directed to the corresponding author.

## Ethics Statement

The studies involving human participants were reviewed and approved by Guangzhou Eighth People’s Hospital (GZ8H20171390). The patients/participants provided their written informed consent to participate in this study.

## Author Contributions

LL and XT designed the research protocol. SX and MW performed the research. WaC, FH, FL, PG, XC, and WeC provided reagents/analytical tools. MW, SX, LL, and XT wrote the manuscript. All authors contributed to the article and approved the submitted version.

## Funding

This work was supported by Guangzhou Basic Research Program on People’s Livelihood Science and Technology [202002020005] and Chinese 13th Five-Year National Science and Technology Major Project [2018ZX10302103-002 and 2018ZX10302104-001-007].

## Conflict of Interest

The authors declare that the research was conducted in the absence of any commercial or financial relationships that could be construed as a potential conflict of interest.
