# Supplementary material for: The Association of TLR2, TLR3, and TLR9 Gene Polymorphisms With Susceptibility to Talaromycosis Among Han Chinese AIDS Patients in Guangdong
Source: Front Cell Infect Microbiol. 2021 Mar 11;11:625461. doi: 10.3389/fcimb.2021.625461 (PMC7991721; doi:10.3389/fcimb.2021.625461)
Supplement: Supplementary file 1 [file Table_1.docx]

**Supplementary Table 1**

Upstream and downstream primers used for the PCR amplification

| Gene | SNP ID | Forward 5′-3′ and reverse 3′-5′ PCR primers | PCR product(bp) |
| --- | --- | --- | --- |
| TLR2 | rs7682814 | F:ACGTTGGATGTGGTAGAGACAGGGTTTTGC | 94 |
|  |  | R:ACGTTGGATGCTAGGCTGGTGGATCACTTG |  |
|  | rs76112010 | F:ACGTTGGATGACGAGACTCCATCTCAGAAA | 120 |
|  |  | R:ACGTTGGATGACAAAGCTATTAGCACGTCC |  |
|  | rs5743708 | F:ACGTTGGATGCCAGGTAGGTCTTGGTGTTC | 104 |
|  |  | R:ACGTTGGATGTTCTTCTGGAGCCCATTGAG |  |
|  | rs121917864 | F:ACGTTGGATGGTCAATGATCCACTTGCCAG | 99 |
|  |  | R:ACGTTGGATGGGAGCTGGAGAACTTCAATC |  |
|  | rs1339 | F:ACGTTGGATGGTCCTATTCCAGCCCTAGTG | 101 |
|  |  | R:ACGTTGGATGCTTCGTTATTTGGTGGCCTG |  |
|  | rs11938228 | F:ACGTTGGATGTAGGGACATGCCCATATAGG | 109 |
|  |  | R:ACGTTGGATGTAGTGTGTTGCTGGTATAGG |  |
|  | rs11935252 | F:ACGTTGGATGGGGCTTTCCTGTAGTCATAC | 119 |
|  |  | R:ACGTTGGATGTCAAAGACAGCAGAAAAAGG |  |
|  | rs7656411 | F:ACGTTGGATGCCTTTAAATTACTGTGTATC | 113 |
|  |  | R:ACGTTGGATGGTACATGTGAGCTAAATAG |  |
|  | rs3804099 | F:ACGTTGGATGGATCTACAGAGCTATGAGCC | 95 |
|  |  | R:ACGTTGGATGCTGCTTCATATGAAGGATCAG |  |
| TLR4 | rs11536891 | F:ACGTTGGATGCCTGATAGGGATACATAGGG | 100 |
|  |  | R:ACGTTGGATGTGGGTGTGTTTCCATGTCTC |  |
|  | rs1927911 | F:ACGTTGGATGCAGACCTTCCTTAGTCATGG | 99 |
|  |  | R:ACGTTGGATGCATCACTTTGCTCAAGGGTC |  |
|  | rs7037117 | F:ACGTTGGATGTCTATCCCAAGATCGGTTCC | 106 |
|  |  | R:ACGTTGGATGAAGAGGCTAGAAGAAGATAG |  |
|  | rs5030728 | F:ACGTTGGATGCTTGGCTACCAACTAACAAC | 117 |
|  |  | R:ACGTTGGATGGCCTTGGATCAAGTTTAGCC |  |
|  | rs10116253 | F:ACGTTGGATGGGCATGCTCCAGAGCAAATC | 114 |
|  |  | R:ACGTTGGATGTGTGTTCTTGATGTTCTGGC |  |
|  | rs7856729 | F:ACGTTGGATGTGGGCCTGATTTGTTCACTG | 99 |
|  |  | R:ACGTTGGATGTTACAGCTGTAAGAGCCAGG |  |
|  | rs11536889 | F:ACGTTGGATGACCCCATTAATTCCAGACAC | 107 |
|  |  | R:ACGTTGGATGTTTCCTGTTGGGCAATGCTC |  |
|  | rs1554973 | F:ACGTTGGATGAGCCACGCTACTCAAAACAC | 96 |
|  |  | R:ACGTTGGATGAAGAGGAGAGAAAGACACCG |  |
|  | rs4986790 | F:ACGTTGGATGCACACTCACCAGGGAAAATG | 105 |
|  |  | R:ACGTTGGATGAGCATACTTAGACTACTACC |  |
|  | rs2737191 | F:ACGTTGGATGCAGCATATGCATTACCTGCC | 111 |
|  |  | R:ACGTTGGATGGGACCCATAATCAGTCAGTC |  |
|  | rs4986791 | F:ACGTTGGATGAGGTTGCTGTTCTCAAAGTG | 118 |
|  |  | R:ACGTTGGATGAGCCCAAGAAGTTTGAACTC |  |
|  | rs11536878 | F:ACGTTGGATGTTCTTGACTACCCACCACAG | 117 |
|  |  | R:ACGTTGGATGGCGACATATAACAGTAGGTG |  |
| TLR9 | rs187084 | F:ACGTTGGATGTTACTATGTGCTGGGCACTG | 108 |
|  |  | R:ACGTTGGATGTATTCCCCTGCTGGAATGTC |  |
|  | rs352140 | F:ACGTTGGATGATAAGCTGGACCTCTACCAC | 99 |
|  |  | R:ACGTTGGATGTGGCTGTTGTAGCTGAGGTC |  |
